# Retracted: Clinical Significance of Resistin Expression in Osteoarthritis: A Meta-Analysis

**DOI:** 10.1155/2020/8709036

**Published:** 2020-12-21

**Authors:** BioMed Research International

BioMed Research Internationalhas retracted the article titled “Clinical Significance of Resistin Expression in Osteoarthritis: A Meta-Analysis” [1]. This article is one of a series of very similar meta-analyses written by different authors that were published in 2014 and 2015, characterized by the use of particular phrases [2]. The articles have the same structure, with the figures in the same order. The appearance of the figures and parts of the text are also similar.

The statistical tests in Figure 6 are underpowered, because they have less than 10 studies.

The original files submitted by the authors were edited by MedChina, a company previously alleged to be involved in the sale of articles [3]. The authors could not be contacted.
